# Elevated FPR confers to radiochemoresistance and predicts clinical efficacy and outcome of metastatic colorectal cancer patients

**DOI:** 10.18632/aging.101864

**Published:** 2019-03-21

**Authors:** Qing-Gen Chen, Lei Zhang, Fan Sun, Shu-Qi Li, Xia-Hong You, Yu-Huan Jiang, Wei-Ming Yang, Qiong-Hui Zhong, Xiao-Zhong Wang, Hou-Qun Ying

**Affiliations:** 1 Department of Clinical Laboratory, Jiangxi Province Key Laboratory of Laboratory Medicine, The Second Affiliated Hospital of Nanchang University, Nanchang 330006, Jiangxi, China; * Equal contribution

**Keywords:** fibrinogen to pre-albumin ratio, systematic inflammation, primary tumor sidedness, metastatic colorectal cancer

## Abstract

Association of chronic inflammation, primary tumor sidedness, adjuvant therapy and survival of metastatic colorectal cancer (mCRC) remains unclear. Circulating inflammatory cell, fibrinogen (Fib), albumin (Alb), pre-albumin (pAlb), Alb/Fib (AFR) and Fib/pAlb (FPR) were detected, and clinical outcome was obtained to determine the predictive, prognostic and monitoring roles of them in discovery and validation cohort. We found that elevated FPR, low AFR and poor survival was observed in right-sided mCRC comparing to the left-sided disease, elevated FPR harbored the highest areas under curve to independently predict poor progression-free survival and overall survival in overall and left-sided mCRC case in two cohorts. No survival difference was examined between the two-sided patients in subgroups stratified by FPR. Radiochemoresistance was observed in high FPR case. However, the patient could benefit from bevacizumab plus radiochemotherapy. Low FPR patient showed the best survival with treatment of palliative resection plus radiochemotherapy. Moreover, circulating FPR was significantly increased ahead imaging confirmed progression and it reached up to the highest value within three months before death. Additionally, c-indexes of the prognostic nomograms including FPR were significantly higher than those without it. These findings indicated that FPR was an effective and independent factor to predict progression, prognosis and to precisely identify the patient to receive optimal therapeutic regimen.

## Introduction

Colorectal cancer (CRC) is a kind of molecular heterogeneous disease that undergo a variety of clinical courses and possess diverse therapeutic responses [1–3]. It has developed to be the third most common malignancy in women and the fourth among men, and it is the fifth cause of cancer-related death in China [4]. Emerged early diagnosed test, painless colonoscopy, oncotarget and immune checkpoint therapy have contributed to improving screening, diagnosis and survival of the disease [5–7], nevertheless, the early diagnostic rate and outcome of CRC patient are still unsatisfactory. The incidence and mortality rates of CRC were rapidly increased in developing countries including China [8], and high case-fatality ratio (14.0%) and mortality/incidence ratio (52.1%) were observed in the past decade in China [9]. Therefore, it is urgent for us to explore the effective tools to precisely screening and discriminating the high-risk individual and to directly manage of the recurrent and metastatic patients.

In recent decades, a continued rightward of CRC was reported by several population-based epidemiological studies [10,11], and the different origin of development as well as distinct anatomic structure were observed in colon and rectal cancer [12]. For this, accumulating studies paid more attention to the role of primary tumor sidedness in this disease. It’s widely recognized an obvious clinical and biological distinction within left- and right-sided CRC [13–15]. However, the clinical utility of this distinction remains unclear in adjuvant therapy response, recurrence and clinical outcome within the right- and light-sided localized CRC. Furthermore, the debates in terms of the clinical efficacy of bevacizumab plus adjuvant chemoradiotherpay within metastatic CRC are ongoing. Cetuximab plus fluorouracil based chemotherapy was commonly recommended and accepted for treatment of *KRAS* wild-type metastatic CRC (mCRC) patient, whereas the left-sided individual was reported more responsive to the therapeutic regimen [16,17], and the survival of left-mCRC individual was extremely superior to the right patients [15,18]. However, the leading cause of this distinct response remains unknown, and other unmeasured confounding factors that would interfere with the regimen efficacy should be considered within the two-sided mCRC.

Systematic inflammation has been reported as an obvious hallmark within CRC [19,20]. It confers to trigger mutation of oncogene and to form a pre-metastatic niche in secondary organs and tissue sites to promote onset and subsequent metastasis of the cancer [21]. Meanwhile, due to the host response and progression of the disease, malnutrition is another significant determinant in mCRC, and it leads to total extended hospital stay time and pro-treatment poor prognosis and life quality of the patients [22,23]. Albumin (Alb) to fibrinogen (Fib) ratio (AFR) and Fib to pre-albumin (pAlb) ratio (FPR), two novel effective indicators for both chronic inflammation and nutrition status, were significantly related to overall survival (OS) within the II-III stage patient in our previous study [24]. Until now, the role of two indicators within the advanced patients undergoing palliative resection, adjuvant chemoradiotherpay or cetuximab and bevacizumab target therapy remains unknown. Thus, a plausible hypothesis in our study is that underlying chronic inflammation and nutritional status represented by AFR and FPR may involve in clinical efficacy of the common therapeutic regimen and outcome of the two-sided mCRC patient.

In present study, we investigated the association between FPR, AFR, localization of the primary tumor, clinical therapy and survival within discovery (302 left-sided and 128 right-sided mCRC patients) and validation cohort (46 left-sided and 31 right-sided mCRC patients). We found that significant severe chronic inflammation and malnutrition represented by FPR in right- and left-sided mCRC contributed to radiochemoresistance, resulting in poor response and survival in both sides of mCRC patient. Circulating FPR was an effective, economical and practice indicator to stratify the patient to receive the optimal therapeutic regimen, and to predict progression and survival of left-sided mCRC patient.

## RESULTS

### Baseline characteristics of the patients

**Figure 1:**
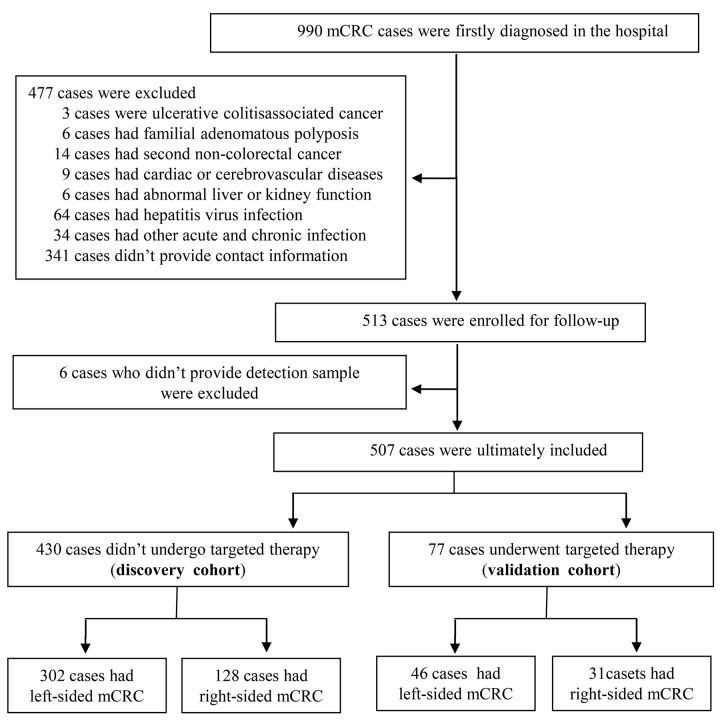
Flow diagram of eligible cases selection in present study.

Overall, a total of 990 firstly diagnosed mCRC patients from November 2011 to May 2015 were prospectively identified in present study, discovery cohort including 430 mCRC patients who didn’t receive targeted therapy and validation cohort containing 77 mCRC cases undergoing bevacizumab plus radiochemotherapy were enrolled as eligible patients according to the inclusion and exclusion criteria. The detail screening flow diagram of eligible patients and the baseline characteristics as well as laboratory detection were described in Figure 1 and Supplementary Table 1. All of the patients were firstly diagnosed mCRC, and liver, peritoneum and multiple site metastasis within the patients accounted for 51.16%, 20.47% and 11.63% in discovery cohort, respectively. Due to poverty or oldness, 20.47% of the patient did not receive any treatment after the firstly diagnosis. Sixty-four and 152 cases received palliative resection and adjuvant radiochemotherapy alone. Only 126 and 77 cases received palliative surgery plus radiochemotherapy and bevacizumab plus radiochemotherapy, respectively. The median AFR and FPR in mCRC patient were 11.13 (IQR: 8.83-14.05) and 23.18 (IQR: 15.31-43.08), 10.42 (IQR: 8.32-12.61) and 25.5 (IQR: 14.57-36.83) in the two cohorts, respectively. After the three years’ follow-up, the median PFS and OS were 8.0 (IQR: 4.0-13.0), 12.0 (IQR: 5.0–23.0) months in discovery cohort and 14.5 (IQR: 8.0-20.0), 16.0 (IQR: 10.0-27.0) in validation cohort, respectively.

### Systematic inflammation and survival in discovery cohort

**Figure 2:**
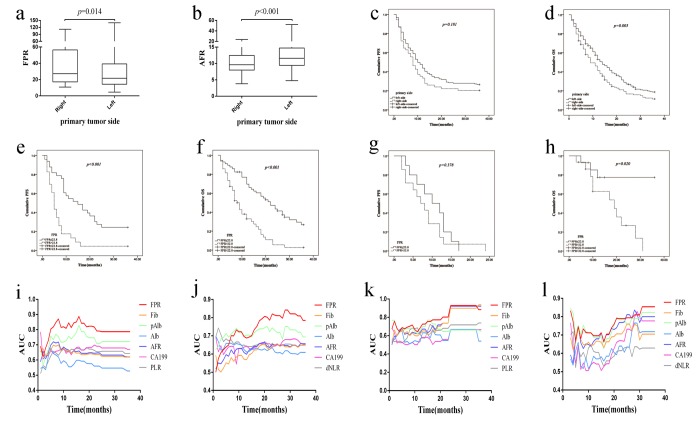
**Comparison and evaluation of FPR, AFR and survival between right- and left-sided mCRC patient in two cohorts.** (**a**) FPR in discovery cohort; (**b**) AFR in discovery cohort; (**c**) Kaplan-Meier curve of primary tumor sidedness for PFS; (**d**) Kaplan-Meier curve of primary tumor sidedness for OS; (**e**) Kaplan-Meier curve of FPR for PFS of left-sided mCRC patient in discovery cohort; (**f**) Kaplan-Meier curve of FPR for OS of left-sided mCRC patient in discovery cohort; (**g**) Kaplan-Meier curve of FPR for PFS of left-sided mCRC patient in validation cohort; (**h**) Kaplan-Meier curve of FPR for OS of left-sided mCRC patient in validation cohort; (**i**) Time-dependent ROC analysis for PFS of left-sided mCRC patient in discovery cohort; (**j**) Time-dependent ROC analysis for OS of left-sided mCRC patient in discovery cohort; (**k**) Time-dependent ROC analysis for PFS in validation cohort; (**l**) Time-dependent ROC analysis for OS in validation cohort.

In order to investigate the difference of systematic inflammation between left- and right-sided mCRC, we compared the inflammatory indicators in discovery cohort. As shown from Figure 2a-b and Supplementary Figure 1a-b, elevated FPR (*p*=0.014), Fib (*p*=0.002), PLR (*p*<0.001) and low AFR (*p*<0.001), pAlb (*p*=0.019), Alb (*p*=0.003) and LMR (*p*=0.040) were observed in right-sided mCRC in comparison with the left-sided cancer. However, there was no significant difference of NLR, dNLR, CEA and CA199 between the two-sided diseases. Comparing to left-sided mCRC, the number of dead patient (85.16% *vs.*78.48%, *p*=0.004) was obviously increased in right-sided mCRC and OS (13.0 months *vs*. 9.0 months, *p_log-rank test_*=0.005) within left-sided mCRC was extremely longer than that within the right-sided patient, but not PFS (Figure 2c-d). When left-sided mCRC stratified into left colon and rectum, no significant difference of PFS and OS was observed between the two subgroups (Supplementary Figure 1c-d).

### Prognostic role of chronic inflammatory indicator in discovery cohort

Using X-tile software, the optimal cut-off points based on OS within the overall mCRC patients were identified as 22.8 for FPR, 9.9 for AFR, 3.9 g/L for Fib, 139.8 mg/L for pAlb, 35.7 g/L for Alb, 2.9 for NLR, 3.7 for dNLR, 166.1 for PLR and 1.5 for LMR in discovery cohort (Supplementary Figure 2). According to the optimal cut-off values, the patients were divided into high and low subgroups by each indicator.

In overall population, results of Kaplan-Meier curve, univariate and multivariate Cox regression showed that median survival of mCRC patients with elevated FPR (*p*_log-rank test_<0.001, adjusted HR=1.896, 95%CI=1.097-3.279 for PFS; *p*_log-rank test_<0.001, adjusted HR=1.583, 95%CI=1.042-2.405 for OS) was significantly short comparing to those with low FPR, respectively (Supplementary Figure 3a-b and Supplementary Table 2). Furthermore, median PFS of the patient with high PLR (*p*_log-rank test_<0.001, adjusted HR=1.601, 95%CI=1.106-2.317) was obviously worse than the cases harbored low PLR, and there were significant differences of OS within high and low subgroup stratified by dNLR (*p*_log-rank test_<0.001, adjusted HR=1.629, 95%CI=1.298-2.044), Fib (*p*_log-rank test_<0.001, adjusted HR=1.344, 95%CI=1.024-1.764) and pAlb (*p*_log-rank test_=0.002, adjusted HR=0.478, 95%CI=0.321-0.711), respectively (Supplementary Table 2).

In left-sided mCRC patients, elevated FPR (*p*_log-rank test_<0.001, adjusted HR=2.254, 95%CI=1.167-4.354 for PFS; *p*_log-rank test_<0.001, adjusted HR=1.769, 95%CI=1.066- 2.935 for OS), PLR (*p*_log-rank test_<0.001, adjusted HR=1.600, 95%CI=1.052-2.234 for PFS), dNLR (*p*_log-rank test_<0.001, adjusted HR=1.726, 95%CI=1.294-2.303 for OS), Fib (*p*_log-rank test_<0.001, adjusted HR=1.934, 95%CI=1.153-3.243 for PFS; *p*_log-rank test_<0.001, adjusted HR=1.428, 95%CI=1.017-2.007 for OS), and low AFR (*p*_log-rank test_<0.001, adjusted HR=0.568, 95%CI=0.340-0.951 for PFS), Alb (*p*_log-rank test_=0.001, adjusted HR=0.603, 95%CI=0.370-0.983 for PFS; *p*_log-rank test_<0.001, adjusted HR=0.716, 95%CI=0.522-0.982 for OS) and pAlb (*p*_log-rank test_=0.004, adjusted HR=0.517, 95%CI=0.322-0.831 for OS) were associated with poor survival in Kaplain-Meier curve and Cox regression (Figure 2e-f and Supplementary Table 3).

Comparing to high pAlb patients, poor OS was observed in the patients harbored low pAlb (*p*_log-rank test_=0.044) in right-sided mCRC individuals. However, there was no significant association between them when it was adjusted by the other factors. The other inflammatory indicators were not related to the survival of right-sided mCRC patients (Supplementary Figure 3c-d and Supplementary Table 4).

### Prognostic role of FPR in validation cohort

In overall population, the median PFS (*p*_log-rank test_=0.047, crude HR=1.914, 95%CI=1.022-3.583) and OS (*p*_log-rank test_=0.001, crude HR=4.473, 95%CI= 1.648-12.141) within high FPR mCRC patient were obviously worse than the low FPR patient (Supplementary Figure 3e-f). Meanwhile, FPR was still significantly associated with OS (adjusted HR=4.206, 95%CI=1.159-15.266) when it was adjusted by the other confounding factors (Supplementary Table 5). In Kaplan-Meier curve and Cox regression, significant association was also observed between elevated FPR and OS in left-sided mCRC patient (*p*_log-rank test_=0.020, crude HR=4.112, 95%CI=1.126-15.015) (Figure 2g-h and Supplementary Table 6). However, no significant association was observed between FPR and survival of right-sided mCRC patient in validation cohort (Supplementary Table 7).

### Predicted efficacy of FPR in two cohorts

Time-dependent ROC curve was used to evaluate the predicted efficacy of FPR in mCRC patient, and area under the curve (AUC) was selected as the common tool to compare the difference between them. The AUCs of FPR were 0.749 for PFS and 0.740 for OS in overall discovery cohort (Supplementary Figure 4a-b), and all of them were obviously higher than Fib, pAlb, Alb, AFR, PLR, dNLR and CA199, respectively. In left-sided mCRC individual, the first and second highest AUCs of the indicator predicting survival of the patients was FPR (AUC=0.787 for PFS, AUC=0.785 for OS) and pAlb (AUC=0.721 for PFS, AUC=0.694 for OS) (Figure 2i-j). However, the predicted efficacy of pAlb was significantly inferior to FPR. Unfortunately, the predicted AUC of FPR was lower than the other indicators, and CA199 harbored the highest AUC for predicting PFS (AUC=0.822) and OS (AUC=0.739) in right-sided mCRC (Supplementary Figure 4c-d).

In validation cohort, AUC of FPR was 0.884 for predicting PFS and it was the same to Fib and AFR, but it was significantly higher than the other indicators (Figure 2k). Even more intriguingly, AUC of FPR reached up to 0.854 and it was the best indicator to forecast OS in overall population (Figure 2l). Meanwhile, the indicators that harbored the first and second highest AUCs for predicting OS were AFR (AUC=0.877) and FPR (AUC=0.868) in left-sided mCRC patients, and there was no significant difference between them (Supplementary Figure 4e). FPR (AUC=0.813) and CA199 (AUC=0.798) harbored the higher AUCs for predicting OS in right-sided patients (Supplementary Figure 4f). Due to insufficient data, AUC of FPR for predicting PFS in right- or left-sided patients wasn’t calculated.

### FPR and clinical baseline characteristics

**Figure 3:**
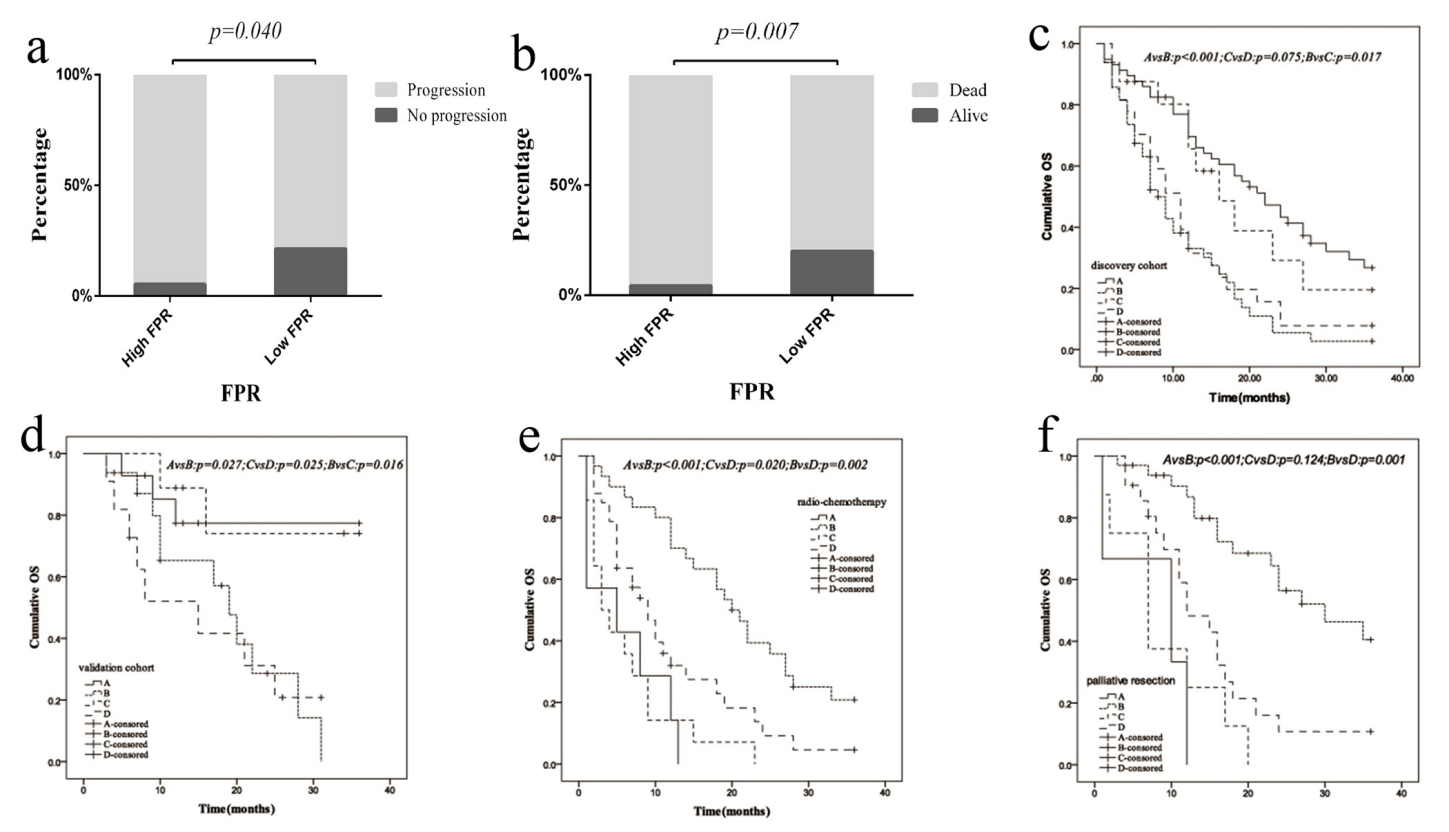
**Relationship between FPR, clinical characteristics and primary tumor sidedness as well as clinical therapeutic efficacy in present study.** (**a**) progression status in discovery cohort stratified by FPR ; (**b**) death status in discovery cohort stratified by FPR; (**c**) overall survival difference between left- and right-sided CRC cases stratified by FPR in discovery cohort; (**d**) overall survival difference between left- and right-sided CRC cases stratified by FPR in validation cohort; (**e**) overall survival difference between high- and low-FPR patients in radiochemotherapy subgroup (no palliative resection); (**f**) overall survival difference between high- and low-FPR patients in palliative resection subgroup. Abbreviation within in Panels 3c-d: A: left-sided patients with low-FPR; B: left-sided mCRC patients with high-FPR; C: right-sided patients with low-FPR; D: right-sided mCRC patients with high-FPR. Abbreviation within in Panels 3e-f: A: low-FPR mCRC patients without radiochemotherapy; B: low-FPR mCRC patient with radiochemotherapy; C: high-FPR mCRC patient without radiochemotherapy; D: high-FPR mCRC patient with radiochemotherapy.

The association between FPR and the baseline characteristics was examined in the two cohorts. The numbers of mCRC case with disease progression (overall population: 94.59% *vs.* 78.57%, *p*=0.040; left-sided mCRC: 95.65% *vs.* 75.76%, *p*=0.047) and death (overall population: 86.84% *vs.* 65.75%, *p*=0.007; left-sided mCRC: 85.71% *vs.* 66.67%, *p*=0.006) within high FPR subgroup were significant higher than the low group only in overall and left-sided mCRC in discovery cohort (Figure 3a-b). Moreover, the number of death in high FPR subgroup was significant higher than the low group in overall and left-sided mCRC (overall population: 72.00% *vs.* 21.74%, *p*<0.001; left-sided mCRC: 73.33% *vs.* 21.43%, *p*=0.009) in validation cohort. However, no obvious association was examined between FPR and the other characteristics in two cohorts.

### FPR, primary tumor sidedness and clinical therapeutic regimen

In present study, the relationship between FPR, primary tumor sidedness and therapeutic regimen was investigated to explore the optimal treatment for mCRC patient. The median OS of low and high FPR mCRC patient were 18.0 and 8.0 months, 20.0 and 7.0 months in both overall population and left-sided subgroups of discovery cohort, and the median OS was 16.0 and 10.0 months, 14.0 and 10.0 months in the two subgroups in validation cohort, respectively. Meanwhile, we also observed the survival of left-sided mCRC patients was obviously longer than that of right-sided individuals in discovery cohort (*p*_log-rank test_=0.005 for OS). Moreover, the median OS of left-sided mCRC patient with high FPR was obviously worse than right-sided patients harbored low FPR in discovery (*p*_log-rank test_=0.017) and validation (*p*_log-rank test_=0.016) cohort (Figure 3c-d). Median OS of high FPR cases was extremely poor in comparison with low FPR patients in both radiochemotherapy (*p*_log-rank test_=0.002) and palliative resection plus radiochemotherapy subgroup (*p*_log-rank test_=0.001) (Figure 3e-f). However, no survival difference was examined between left- and right- sided mCRC individual harbored low (discovery cohort: *p_log-rank test_*=0.492, validation cohort: *p_log-rank test_*=0.863) or high FPR (discovery cohort: *p_log-rank test_*=0.446, validation cohort: *p_log-rank test_*=0.792) in the two cohorts (Figure 3c-d).

**Figure 4:**
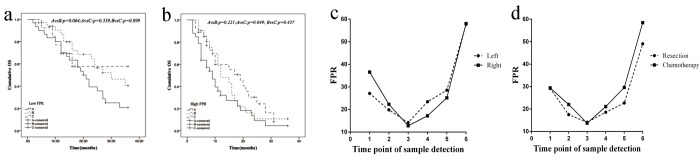
**Survival comparison of the patient received different treatments in the high and low FPR subgroups and the dynamic change of FPR during the treatment.** (**a**) Kaplan-Meier curve of OS within the patient received different treatment in low-FPR subgroup; (**b**) Kaplan-Meier curve of OS within the patient received different treatment in high-FPR subgroup; (**c**) dynamic change of FPR in left- and right-sided mCRC patients; (**d**) dynamic change of FPR of mCRC patient undergoing chemotherapy or palliative resection Abbreviation within in Panels 4a-b: A: radiochemotherapy; B: palliative resection plus radiochemotherapy; C: bevacizumab plus radiochemotherapay. Abbreviation within in Panels 4c-d: 1: the diagnostic time; 2: one month after the first treatment; 3: regular examination without disease progression; 4: one month before disease progression; 5: time of imaging confirmed progression; 6: within three months before death.

In low FPR subgroup, the survival of radiochemotherapy treated mCRC patient was significantly longer than those without the treatment in both palliative surgery (*p*_log-rank test_<0.001) and non-surgery subgroup (*p*_log-rank test_<0.001) (Figure 3e-f). In the same time, the survival of palliative resection plus radiochemotherapy treated mCRC patient was superior to the patients undergoing radiochemotherapy (*p*_log-rank test_=0.064). However, no survival difference was observed between palliative resection plus radiochemotherapy treated mCRC patients and the patients undergoing bevacizumab plus radiochemotherapy *p_log-rank test_*=0.899 (Figure 4a).

In high FPR subgroup, the survival was gradually decreased in mCRC patient treated with bevacizumab plus radiochemotherapy, palliative resection plus radiochemotherapy and radiochemotherapy alone (Figure 4b). OS of the patient treated with radiochemotherapy was superior to the non-treated case (*p_log-rank test_* =0.020) (Figure 3e). The survival of palliative resection plus radiochemotherapy treated mCRC patient was equal to the patient treated with palliative resection or radiochemotherapy alone (palliative resection: *p_log-rank test_*=0.124, radiochemotherapy: *p_log-rank test_*=0.221). Whereas, the significant survival difference was observed between bevacizumab plus radiochemotherapy and radiochemotherapy treated mCRC patient (*p_log-rank test_* =0.049) (Figure 4b).

### Role of FPR in predicting progression of mCRC patients

According to the inclusion criteria and willing of the patient, seventeen palliative resection and thirty-four radiochemotherapy treated mCRC patients were included to investigate the role of FPR in predicting progression of mCRC patients. Each of them provided detected sample in each time point, and all of them were progressed and dead in the follow-up period. As shown from Figure 4c-d, circulating FPR was extremely decreased after the first-treatment, and it reduced significantly to the lowest value in the following two-three months in subgroups stratified by treatment or primary tumor sidedness (all *p*<0.05). Furthermore, it was significantly increased in the time of one month before clinical imaging confirmed progression compared with the lowest point (*p*<0.05), and it reached up to the highest value within three months before death (*p*<0.01).

### Assessment of FPR contained prognostic nomogram in overall mCRC cases

**Figure 5:**
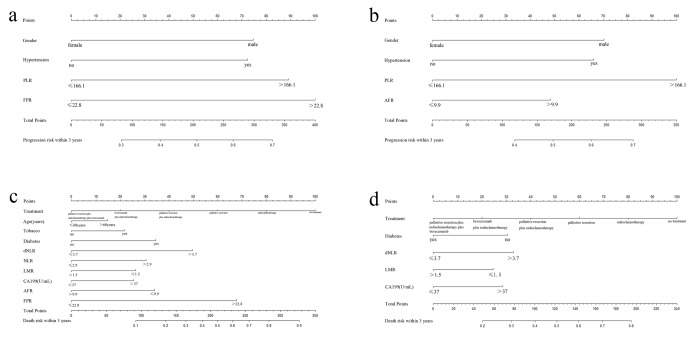
**Prognostic nomograms with or without FPR for predicting survival of mCRC patient.** (**a**) nomogram including FPR for predicting 3 years’ PFS; (**b**) nomogram without FPR for predicting 3 years’ PFS; (**c**) nomogram including FPR for predicting 3 years’ OS; (**d**) nomogram without FPR for predicting 3 years’ OS.

Using the significant characteristics and FPR, we established the prognostic nomograms and evaluated progression and death risk of overall mCRC patients within three years. The prognostic nomograms with or without FPR were showed in Figure 5. The c-indexes of prognostic nomograms with FPR were 0.65 for three years’ progression and 0.74 for three years’ OS. On the contrary, c-indexes of the nomograms without FPR predicting three years’ PFS and OS were only 0.62 and 0.69, respectively. Moreover, the predicted efficacy of the nomogram including FPR was significantly higher than that without FPR in predicting both 3 years’ progression (*p*<0.05) and death (*p*<0.01).

## DISCUSSION

Despite high risk of disease progression triggered by chronic inflammation and malnutrition [25–28], the prognostic, predictive and monitoring role of FPR in left- and right-sided mCRC is not defined. In present study, we found a significant difference of systematic inflammation between left- and right-sided mCRC patient, high FPR was superior to other inflammatory indicators to independently and effectively predict poor PFS and OS for overall mCRC patients, especially for left-sided mCRC individuals. Moreover, FPR could stratify the patient to achieve maximum benefit from the optimal therapeutic regimen. Circulating dynamic FPR was better than common imaging detection to monitor the progression of the disease after the first treatment. In addition, it could improve the prognostic nomograms to predict the survival of mCRC patient.

It has been well known that Fib, Alb and pAlb are not only commonly recognized as a vital factor in coagulation cascade reaction and as a useful indicator to imply nutrition status, respectively, but also can effectively respond to para-inflammation and systematic inflammation in cancer [29–32]. Hyperfibrinogenemia and hypoalbuminemia are usually detected in CRC patients, particularly in mCRC individual [32,33]. Several studies have reported that circulating AFR and FPR can predict the survival of malignancies such as non-small cell lung cancer, esophageal cancer and hepatocellular carcinoma as well as gastric cancer [34–37]. In present study, severe systematic inflammation represented by FPR and other inflammatory indicators was observed in right-sided mCRC comparing to the left-sided case [15,38], which consisted with the report by Patel M and McSorley ST [39]. More progression and death cases were found in overall and left-sided high FPR mCRC patients, and elevated FPR was significantly associated with poor survival of the overall and left-sided patients in the two cohorts, illustrating that FPR wasn’t correlated with the survival of right-sided mCRC cases, but was an independent prognostic factor in predicting clinical outcome of left-sided mCRC patient. AUCs of FPR for predicting PFS and OS were the highest in overall and left-sided mCRC patients in two cohorts, demonstrating that FPR was the most efficient indicator to predict the prognosis of the patient.

**Figure 6:**
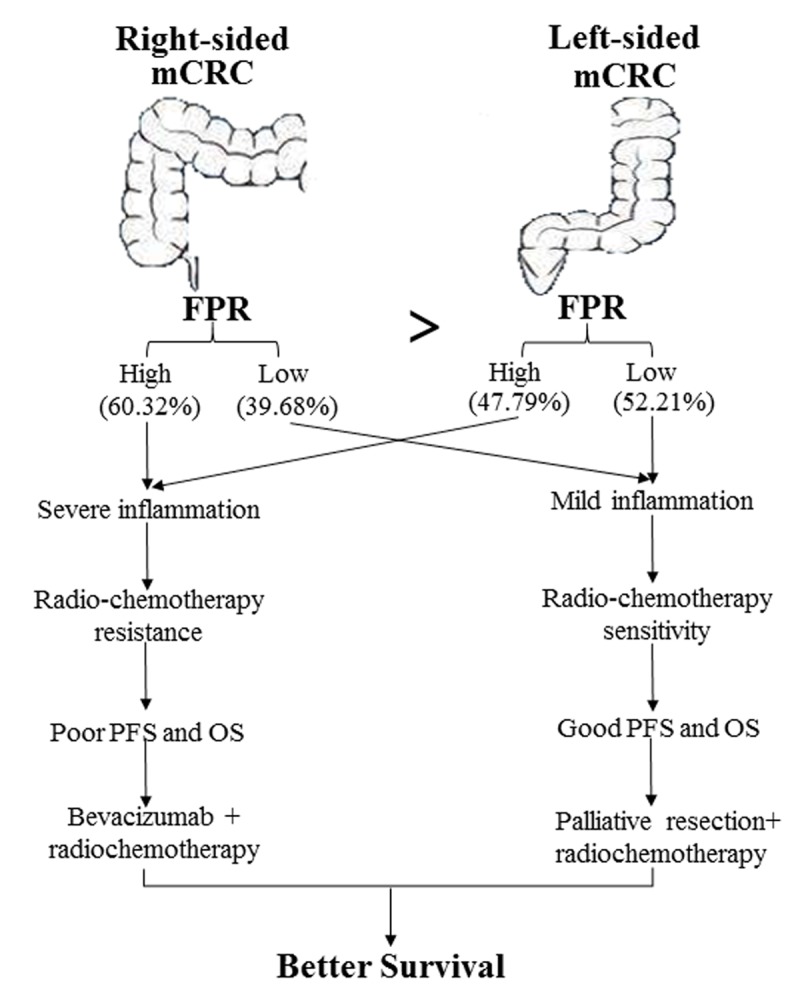
Diagram depicting the cause of survival difference between left- and right-sided mCRC and the optimal common treatment selection for the patients according to FPR.

Nowadays, left- and right-sided CRC have been known as two distinct diseases, for significant differences in mutation spectrum of oncogene and anti-oncogene and prognosis of the two-sided diseases [12,40–42]. In our study, we confirmed the previous findings that the right-sided mCRC patients’ survival was inferior to the left-sided cases in discovery cohort. We also found poor survival within high FPR patients in comparison with the low FPR cases in both right- and left-sided patients. OS of right-sided low FPR mCRC patients was superior to the left-sided high FPR cases in the two cohorts, indicating that clinical outcome of right-sided patients was not completely inferior to the left-sided cases, and FPR could be considered as an important stratified factor to predict prognosis of mCRC patients. To our surprise, there was no different survival between the two sides in low or high FPR subgroup, revealing that FPR was an important confounding factor to impact the prognostic role of primary tumor sidedness in mCRC patients, and survival of the patient was not associated with primary tumor sidedness, but was related to the severity of chronic inflammation. Clinical outcome of low FPR patient was superior to the high FPR cases in radiochemotherapy and palliative resection plus radiochemotherapy subgroups, and chemotherapy treated mCRC patient was equal to the patient without any treatment in palliative surgery subgroup, indicating that only low FPR mCRC patients could benefit from the common treatments, and high FPR might confer to radiochemoresistance in both right- and left-sided mCRC patients (Figure 6).

Our previous study also showed that FPR could stratify the eligible surgical cases who can benefit from adjuvant radiochemotherapy [24]. In present study, the survival of palliative surgery resection plus radiochemotherapy treated low FPR case was the longest in those receiving the common treatments, and was the same to bevacizumab plus radiochemotherapy received patient. On the contrary, survival of bevacizumab plus radiochemotherapy treated high FPR patient was superior to the patient with treatment of adjuvant radiochemotherapy. These findings revealed that palliative resection plus radiochemotherapy and bevacizumab plus radiochemotherapy were the optimal clinical regimens for low and high FPR mCRC patient, respectively (Figure 6). Furthermore, circulating FPR was gradually decreased to the lowest value from the first clinical treatment to the following months without progression, and it was gradually increased from the time of one month before clinical imaging confirmed progression to the last detection before death, elucidating that the indicator could effectively monitor disease progression of the patient. Additionally, the predicted efficacy of prognostic nomogram including FPR was significantly higher than that without the indicator, showing that FPR could improve the prediction efficacy of the prognostic nomogram.

As we known, the interaction between stromal cell, inflammation-related cells such as neutrophil, macrophage and monocyte, inflammatory cytokine and cancer cell remodeled an adapted microenvironment not only to promote progression of the disease but also to involve in resistance to anticancer therapy [43–45]. C-terminal region of Fib γ-chain could interact with CD11b or CD11c/CD18 integrin receptor of inflammatory cell to activate a wide range of inflammatory immune cells such as monocyte, macrophage and neutrophil [46]. These activated immune cells and stromal cells such as CD90^+^ colonic myofibroblast, fibroblast, endothelial cell could secret interleukin-6(IL-6), IL-21 and IL-33 to activate NF-κB and JAK/STAT pathway to promote systematic inflammation of mCRC by regulating differentiation of T helper 17 cell and regulatory T cell [47–49]. Fib, Alb and pAlb were the acute phase reaction proteins in response to the chronic inflammation [50], IL-6 secreted by cancer-associated fibroblast and cancer cell could inhibit pAlb and stimulate Fib, eventually leading to low pAlb and elevated Fib. Meanwhile, IL-6 contributed to cancer chemoresistance by gp130/MAPK/STAT3 mediated activation of transcription factors C/EBPβ/δ, epithelial to mesenchymal transition, overexpression of p-glycoprotein and expansion of cancer stem cells [51]. Moreover, malajusted miR- 155-5p/C/EBPβ/IL6 signaling in tumor-associated macrophage could induce chemoresistance by regulating the IL6R/STAT3/miR-204-5p axis [52]. On the contrary, down-regulated IL-6/GP130 improved 5-fluorouracil-based chemotherapy sensitivity in colon cancer [53]. Consequently, severe cancer chronic inflammation represented by elevated FPR could confer to radiochemoresistance and poor prognosis. Moreover, Severe malnutrition within mCRC patients damaged the patients’ immunologic defense and surveillance [54,55], resulting in unsatisfied response to adjuvant chemotherapy and poor survival of the cancer patient. Additionally, more severe systematic inflammation was observed in right-sided comparing to left-sided patients, and the majority of right-sided mCRC harbored elevated FPR, and low FPR mCRC cases accounted for a small proportion. Therefore, the prognosis of right-sided mCRC patients was inferior to the left-sided cases, and high FPR conferred to poor clinical efficacy of adjuvant chemotherapy, leading to poor survival of the patient.

The prospective study is the first time for us to evaluate the predictive, prognostic and monitoring role of FPR in left- and right-sided mCRC patients. It is also the first time for us to illustrate that severe systematic inflammation involving in radiochemoresistance is the main reason for the survival difference between left- and right-sided mCRC cases. Additionally, dynamic monitoring FPR can predict the disease progression ahead clinical imaging detection, and prognostic nomogram including FPR can efficiently predict the progression and death outcome of mCRC patients. However, the cases in our study are from the single center, and the sample size isn’t large enough. Thus our findings needed to be validated by other prospective study with large sample size from multiple centers. Moreover, in vivo and in vitro experiments are not carried out to furtherly confirm the association between high FPR, radiochemoresistance and poor survival in the mCRC patient.

## Conclusions

In summary, our findings have demonstrated that significant difference of chronic inflammation represented by FPR has an impact on radiochemotherapy sensitivity, resulting in significant survival difference in right- and left-sided mCRC. Elevated FPR is an efficient and independent prognostic factor to predict poor survival of left-sided mCRC patients and to improve the efficacy of its prognostic nomogram. It serves as a readily valuable indicator for monitoring progression and can be considered as a precise stratification factor in future clinical trials aiming to optimize common treatment strategies for the disease.

## MATERIALS AND METHODS

### Population

In our study, a total of 990 firstly diagnosed mCRC patients in the Second Affiliated Hospital of Nanchang University were prospectively identified from November 2011 to May 2015. We screened the eligible case according to the following inclusion and exclusion criteria: 1) the patient should be firstly diagnosed as mCRC by X-ray, CT, MRI or pathological detection; 2) all patients should be free of hereditary polyposis and nonpolyposis CRC, emergency surgery, palliative operation, neoadjuvant chemoradiotherapy, ulcerative colitis-associated cancer ahead the clinical confirmation; 3) the eligible cases were not suffered from other malignancies, recent bacterium and virus infection, autoimmune and hematologic as well as cardiovascular and cerebrovascular disease; 4) liver and kidney function should be normal in all the included patients; 5) all included patients didn’t intake either drugs such as antibacterial agent, non-steroidal anti-inflammatory drug, antiplatelet or anticoagulant drug nor intravenous albumin supplement in recent three months; 6) the eligible patient could provide complete clinical characteristics, and contact information for three years’ follow-up; 7) the patient signed the informed consent and they agreed to collect pre-treatment peripheral, serum, and plasma samples for late-stage detection. The study was approved by the Medical Ethics Committee of the local hospital.

### Clinical baseline characteristics and follow-up

The baseline characteristics such as demographic and clinicopathological data and clinical therapeutic regimen were extracted from the medical record. The tumor location from ileocecal section to the splenic flexure (without it) and the splenic flexure to rectum were considered as right- and left-sided CRC, respectively. Routine three years’ follow-up (one season a time within the first two years, and six months in the third year) were performed by means of email, telephone, and medical record in all eligible patients after the first time treatment and the follow-up deadline was July 2018. Three years’ progression-free survival (PFS) and overall survival (OS) were the main endpoints in present study, the two of them were measured from the first clinical confirmation until the date of local progression or new distant-site metastasis and death from any cause, respectively.

### Sample collection and laboratory detection

In order to investigate the relationship of systematic inflammation, primary tumor location with clinical efficacy and outcome of mCRC patient, the respective two milliliters pre-treatment peripheral blood, serum, and plasma samples were collected from all eligible patients for detection. All the samples were collected at 7:00 to 9:00 am ahead the first clinical confirmed time. Moreover, mCRC patients with both progression and death event were selected to collect these samples in the following each time point (one month after the first treatment, regular examination without disease progression, one month before the progression, time of clinical imaging confirmed progression, within three months before death) and all the detections were completed within two hours after the sample collection.

SYSMEX XE-2100 machine (Sysmex, Tokyo, Japan) with nucleic acid fluorescence staining and laser flow analysis method was used to detect differential white cell count. Plasma Fib was measured by clauss method using SYSMEX CA-7000 machine (Sysmex, Tokyo, Japan). Bromcresol green dye method, immunoturbidimetric assay and electrochemiluminescence immunoassay were selected to examine serum Alb, pAlb, carcinoembryonic antigen (CEA) and carbohydrate antigen 199 (CA199) with machines of OLYMPUS AU5400 (Beckman Coulter, Tokyo, Japan) and SIEMENS ADVIA Centaur CP machine (Siemens, Erlangen, Germany), respectively. The inter- and intra-batch coefficients of variation of immune cell count, Fib, Alb, pAlb, CEA and CA199 kits were less than 5%. According to the detection result, we calculated the following ratios: Alb to Fib ratio (AFR), Fib to pAlb ratio (FPR), neutrophil to lymphocyte ratio (NLR), (total white cell-lymphocyte) to lymphocyte ratio (derived neutrophil to lymphocyte ratio, dNLR), platelet to lymphocyte ratio (PLR), lymphocyte to monocyte ratio (LMR).

### Statistics

Continuous variables with normal and skewed distribution were expressed as mean±standard deviation, median and inter-quartile ranger (IQR), respectively. Chi-square or Fisher’s exact test was used to compare the difference of categorical variable, and continuous variable with skewed distribution was compared by Mann-Whitney U test. The optimal cut-off points of Fib, Alb and pAlb and the ratios replying on three years’ OS were determined by X-tile 3.6.1 software (Yale University, New Haven, CT, USA). The difference of survival rate was calculated using Kaplan-Meier curve with log-rank test. Cox proportional regression with hazard ratio (HR) and 95% confidential interval (CI) was applied to examine the prognostic role of clinical baseline characteristics and laboratory detected indicators in overall, left- and right-sided mCRC patient. Prognostic predictive efficacy of the significant factor was assessed and compared by time-dependent receiver operating characteristic (ROC) curve. The significant characteristics and laboratory markers were used to establish PFS and OS prognostic nomograms, and Harrell’s concordance index (c-index) were selected to evaluate their predicted efficacy. All the statistics were analyzed using SPSS. 22.0 (IBM Corp, Armonk, NY, USA) and R 3.5.1(Institute for Statistics and Mathematics, Vienna, Austria). All analyses were two-sided, and *p<*0.05 was recognized as a statistical significance.

### Ethics approval and consent to participate

The study was approved by the institutional ethics committees of the Second Affiliated Hospital of Nanchang University and all procedures were conducted in accordance with ethical principles.

### Availability of data and materials

The datasets used and/or analyzed during the current study are available from the corresponding author on reasonable request.

## Supplementary Material

Supplementary Figures

Supplementary Tables
