# Supplementary material for: Elevated FPR confers to radiochemoresistance and predicts clinical efficacy and outcome of metastatic colorectal cancer patients
Source: Aging (Albany NY). 2019 Mar 21;11(6):1716–32. doi: 10.18632/aging.101864 (PMC6461181; doi:10.18632/aging.101864)
Supplement: Supplementary Figures [file aging-11-101864-s001.pdf]

SUPPLEMENTARY FIGURES

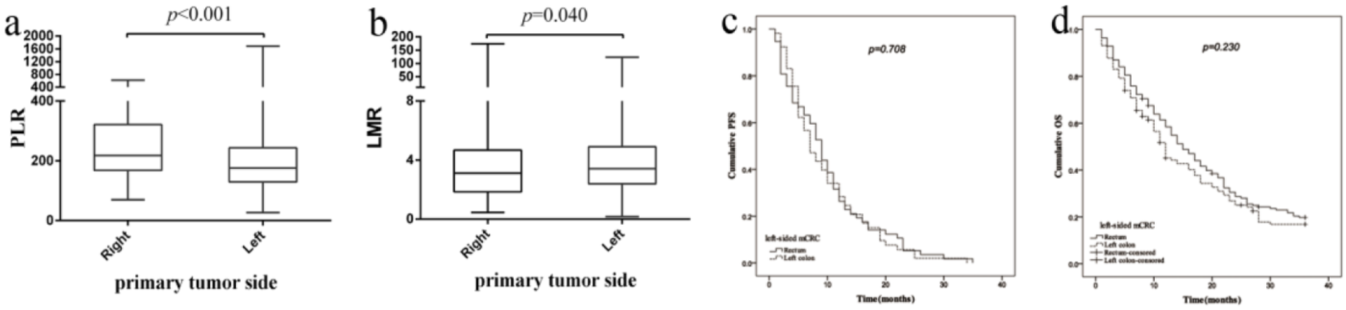

Supplementary Figure 1. Comparison of PLR, LMR and OS between left- and right-sided mCRC patient in discovery cohort. (**a**) PLR in discovery cohort; (**b**) LMR in discovery cohort; (**c**) Kaplan-Meier curve of left colon and rectum for PFS; d: Kaplan-Meier curve of left colon and rectum for OS.

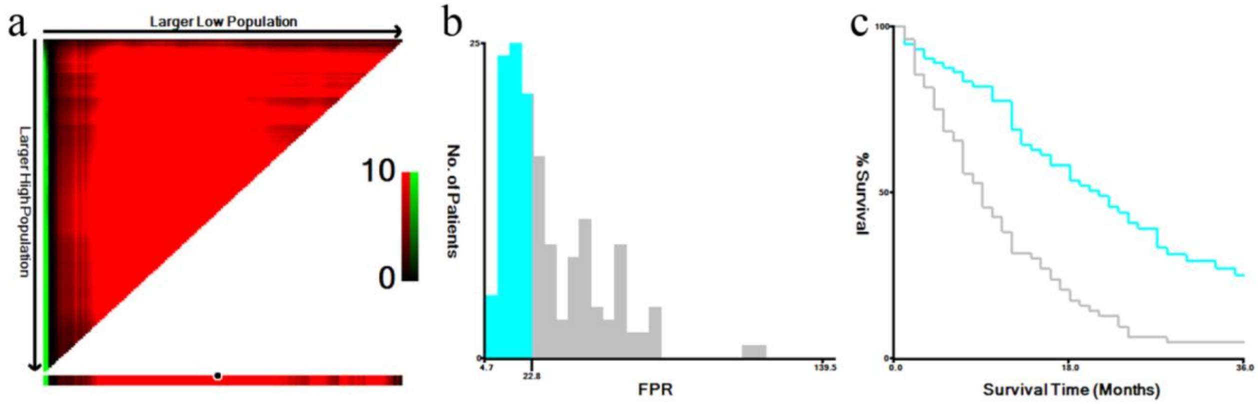

Supplementary Figure 2. The optimal cut-off of pre-treatment FPR in discovery cohort using X-tile software. (**a**) The data were represented graphically in a right-triangular grid where each point represents the data from a given set of divisions. The plots showed the χ^2 log-rank values produced, dividing them into two groups by the cut-off point. The optimal cut-points was determined by locating the brightest pixel on the X-tile plot. The distribution of number of patients was shown on the histogram (**b**) and corresponding populations were displayed on the Kaplan-Meier curve (**c**).

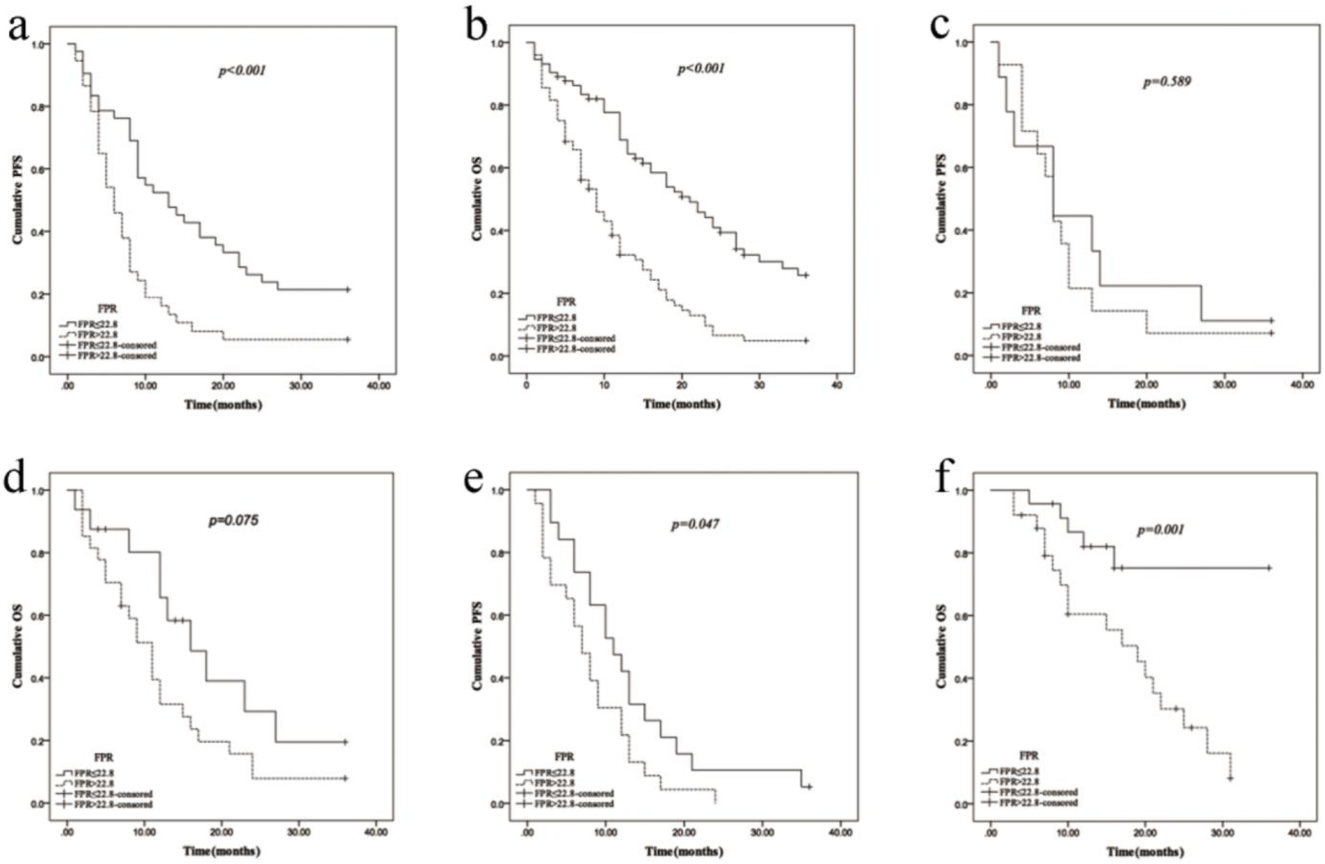

Supplementary Figure 3. Kaplan-Meier curves of FPR for 3 years' PFS and OS in two cohorts. (**a**) Kaplan-Meier curve of FPR for PFS in discovery cohort; (**b**) Kaplan-Meier curve of FPR for OS in discovery cohort; (**c**) Kaplan-Meier curve of FPR for PFS of right-sided mCRC patient in discovery cohort; (**d**) Kaplan-Meier curve of FPR for OS of right-sided mCRC patient in discovery cohort; (**e**) Kaplan-Meier curve of FPR for PFS in validation cohort; f: Kaplan-Meier curve of FPR for OS in validation cohort.

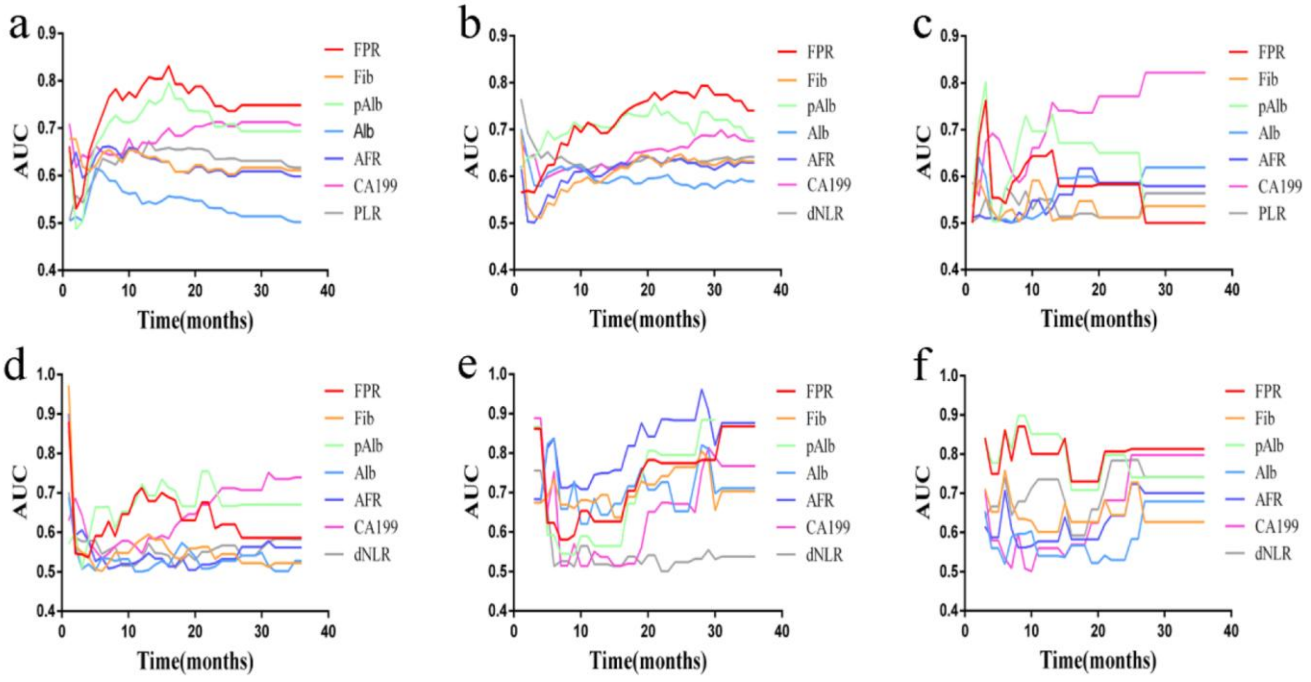

Supplementary Figure 4. Time-dependent ROC analysis of FPR for 3 years' PFS and OS in two cohorts. (**a**)Time-dependent ROC analysis for PFS in discovery cohort; (**b**) Time-dependent ROC analysis for OS in discovery cohort. (**c**) Time-dependent ROC analysis for PFS of right-sided mCRC patient in discovery cohort; (**d**) Time-dependent ROC analysis for OS of right-sided mCRC patient in discovery cohort. (**e**) Time-dependent ROC analysis for OS of left-sided mCRC patient in validation cohort. (**f**) Time-dependent ROC analysis for OS of right-sided mCRC patient in validation cohort.
