# Supplementary material for: Elevated FPR confers to radiochemoresistance and predicts clinical efficacy and outcome of metastatic colorectal cancer patients
Source: Aging (Albany NY). 2019 Mar 21;11(6):1716–32. doi: 10.18632/aging.101864 (PMC6461181; doi:10.18632/aging.101864)
Supplement: Supplementary Tables [file aging-11-101864-s002.pdf]

SUPPLEMENTARY TABLES

Supplementary Table 1. Clinical and pathological characteristics in 507 colorectal cancer patients.

.

Variables	Categories	No. of patients (%)	
		Discovery cohort (430)	Validation cohort (77)
Gender	Male	243(56.51%)	41(53.25%)
	Female	187(43.49%)	36(46.75%)
Age	≤60years	206(47.90%)	51(66.23%)
	>60years	224(52.1%)	26(33.77%)
Tobacco	Yes	88(20.47%)	14(18.18%)
	No	342(79.53%)	63(81.82%)
Alcohol	Yes	71(16.51%)	7(9.09%)
	No	359(83.49%)	70(90.91%)
Hypertension	Yes	60(13.95%)	14(18.18%)
	No	370(86.05%)	63(81.82%)
Diabetes	Yes	30(6.98%)	4(5.19%)
	No	400(93.02%)	73(94.81%)
Tumor side	Left	302(70.23%)	46(59.74%)
	Right	128(29.77%)	31(40.26%)
Metastasis	Liver	220(51.16%)	31(40.26%)
	Lung	24(5.58%)	2(2.60%)
	bone	11(2.56%)	0(0.00%)
	Peritoneu	88(20.47%)	15(19.48%)
	Urogenital system	37(8.60%)	5(6.49%)
	Multiple	50(11.63%)	24(31.17%)
No palliative surgery	No treatment	88(20.47%)	-
	Radiochemotherapy alone	152(35.35%)	41(53.25%)

Palliative surgery	Surgery alone	64(14.88%)	-
	Surgery and radiochemotherapy	126(29.30%)	36(46.75%)
CEA	⩽5 ng/mL	106(24.65%)	19(24.68%)
	>5 ng/mL	292(67.91%)	43(55.84%)
	NA	32(7.44%)	15(19.48%)
CA199	⩽37 U/mL	185(43.02%)	21(27.27%)
	>37 U/mL	212(49.30%)	42(54.55%)
	NA	33(7.68%)	14(18.18%)
NLR(median, IQR)		3.14(2.25-4.60)	3.23(2.41-4.48)
dNLR(median, IQR)		3.57(2.58-5.23)	3.71(2.78-5.08)
LMR(median, IQR)		3.31(2.23-4.86)	2.97(2.22-4.24)
PLR(median, IQR)		189.92(140.26-255.25)	169.37(128.07-224.92)
Fib (median, IQR)	g/L	3.48 (2.90-4.26)	3.87(3.09-4.43)
Alb (median, IQR)	g/L	38.85 (35.10-41.70)	39.83(36.92-44.12)
pAlb (median, IQR)	mg/L	154.00(102.52-199.77)	160.00(111.32-208.14)
AFR (median, IQR)		11.13 (8.83-14.05)	10.42(8.32-12.61)
FPR (median, IQR)		23.18(15.31-43.08)	25.50(14.57-36.83)
Number of death		346(80.47%)	45(58.44%)
PFS (median, IQR)	months	8.0(4.0-13.0)	14.5(8.0-20.0)
OS (median, IQR)	months	12.0(5.0-23.0)	16.0(10.0-27.0)

Abbreviation: Fib: fibrinogen; Alb: albumin; pAlb: pre-albumin; AFR: albumin to fibrinogen ratio; FPR: fibrinogen to pre-albumin ratio; PLR: Platelet to lymphocyte ratio; dNLR: derived neutrophil to lymphocyte ratio; NLR: Neutrophil granulocyte to lymphocyte ratio; LMR: lymphocyte to monocyte ratio; CEA: carcinoembryonic antigen; CA199: carbohydrate antigen 19-9; NA: not available; PFS: progression-free survival; OS: Overall survival; IQR: interquartile ranges.

Supplementary Table 2. Univariate and multivariate analyses of prognostic factors for 3 years' PFS and OS by Cox regression model in discovery cohort.

Variables	Progression-free survival				Overall survival			
	Univariate analysis		Multivariate analysis		Univariate analysis		Multivariate analysis	
	**Crude HR(95% CI)**	***p*-value**	**Adjusted HR(95% CI)**	***p*-value**	**Crude HR(95% CI)**	***p*-value**	**Adjusted HR(95% CI)**	***p*-value**
Sex (male)	1.208(0.876-1.667)	0.249	-	-	1.149(0.928-1.422)	0.203	-	-
Age (>60 years)	1.107 (0.805-1.521)	0.531	-	-	1.488(1.203-1.841)	**<0.001**	1.477(1.159-1.882)	**0.002**
Alcohol (yes)	0.739(0.466-1.172)	0.198	-	-	0.906(0.684-1.199)	0.490	-	-
Tobacco (yes)	0.663(0.437-1.004)	0.052	-	-	0.806(0.619-1.048)	0.107	-	-
Hypertension (yes)	0.859(0.556-1.328)	0.494	-	-	0.829(0.608-1.131)	0.237	-	-
Diabetes (yes)	1.066(0.652-1.744)	0.798	-	-	0.721(0.468-1.110)	0.138	-	-
Primary side (Right)	1.261(0.887-1.795)	0.197	-	-	1.367(1.089-1.716)	**0.007**	-	-
Palliative surgery (Yes)	0.736(0.535-1.014)	0.060	-	-	0.521(0.419-0.648)	**<0.001**	0.511(0.402-0.649)	**<0.001**
Radio-chemotherapy (Yes)	1.111(0.755-1.633)	0.594	-	-	0.568(0.457-0.705)	**<0.001**	0.600(0.473-0.762)	**<0.001**
CEA (>5ng/mL)	1.475(1.020-2.132)	**0.039**	-	-	1.407(1.085-1.825)	**0.010**	-	**-**
CA199 (>37U/mL)	2.170(1.540-3.057)	**<0.001**	2.049(1.446-2.094)	**<0.001**	1.727(1.379-2.164)	**<0.001**	1.678(1.329-2.119)	**<0.001**
PLR(>166.1)	1.749(1.260-2.429)	**0.001**	1.601(1.106-2.317)	**0.013**	1.636(1.306-2.050)	**<0.001**	-	**-**
dNLR(>3.7)	1.620(1.175-2.234)	**0.003**	-	-	1.791(1.448-2.215)	**<0.001**	1.629(1.298-2.044)	**<0.001**
NLR (>2.9)	1.811(1.312-2.500)	**<0.001**	-	-	1.786(1.434-2.223)	**<0.001**	-	**-**
LMR(>1.5)	0.539(0.305-0.953)	**0.034**	-	-	0.470(0.342-0.646)	**<0.001**	-	**-**
Fib (>3.9 g/L)	2.257(1.573-3.239)	**<0.001**	-	-	1.606(1.251-2.062)	**<0.001**	1.344(1.024-1.764)	**0.033**
Alb (>35.7 g/L)	0.726(0.491-1.073)	0.108	-	-	0.648(0.515-0.815)	**<0.001**	-	**-**
pAlb (>139.8mg/L)	0.491(0.302-0.799)	**0.004**	-	-	0.443(0.306-0.640)	**<0.001**	0.478(0.321-0.711)	**<0.001**
AFR (>9.9)	0.541(0.378-0.775)	**0.001**	-	-	0.579(0.452-0.740)	**<0.001**	-	-
FPR (>22.8)	2.326(1.419-3.815)	**0.001**	1.896 (1.097-3.279)	0.022	2.598(1.768-3.816)	**<0.001**	1.583(1.042-2.405)	**0.031**

Abbreviation: HR: hazard ratio; CI: confidence interval; Fib: fibrinogen; Alb: albumin; pAlb: pre-Albumin; AFR: albumin to fibrinogen ratio; FPR: fibrinogen to pre-Albumin ratio; PLR: Platelet to lymphocyte ratio; dNLR: derived neutrophil to lymphocyte ratio; NLR: Neutrophil granulocyte to lymphocyte ratio; LMR: lymphocyte to monocyte ratio; CEA: carcinoembryonic antigen; CA199: carbohydrate antigen 19-9; adjusted HR (95%) was adjusted by sex, age, alcohol, tobacco, hypertension, diabetes, primary side, palliative resection, radio-chemotherapy, CEA, CA199, PLR, dNLR, NLR, LMR.

Supplementary Table 3. Univariate and multivariate analyses of prognostic factors for 3 years' PFS and OS by Cox regression model in left-sided mCRC patient in discovery cohort.

Variables	Progression-free survival				Overall survival			
	Univariate analysis		Multivariate analysis		Univariate analysis		Multivariate analysis	
	Crude HR(95% CI)	*p*-value	Adjusted HR(95% CI)	*p*-value	Crude HR(95% CI)	*p*-value	Adjusted HR(95% CI)	*p*-value
Sex (male)	1.065(0.728-1.559)	0.745	-	-	1.082(0.835-1.402)	0.553	-	-
Age (>60 years)	1.353(0.855-1.812)	0.253	-	-	1.723(1.329-2.234)	**<0.001**	1.723(1.285-2.310)	**<0.001**
Alcohol (yes)	0.775(0.467-1.285)	0.323	-	-	0.878(0.633-1.219)	0.438	-	-
Tobacco (yes)	0.651(0411-1.031)	0.067	-	-	0.753(0.554-1.022)	0.069	-	-
Hypertension (yes)	1.001(0.604-1.658)	0.997	-	-	0.913(0.632-1.319)	0.628	-	-
Diabetes (yes)	0.966(0.544-1.890)	0.966	-	-	0.732(0.426-1.256)	0.258	-	-
Palliative resection (Yes)	0.735(0.505-1.070)	0.108	-	-	0.501(0.383-0.654)	**<0.001**	0.592(0.441-0.794)	**<0.001**
Radio-chemotherapy (Yes)	1.258 (0.788-2.009)	0.336	-	-	0.611(0.468-0.798)	**<0.001**	0.716(0.528-0.972)	**0.032**
CEA (>5ng/mL)	1.393(0.915-2.120)	0.122	**-**	**-**	1.274(0.940-1.727)	0.118	-	-
CA199 (>37U/mL)	1.977(1.332-2.935)	**0.001**	1.940(1.298-2.900)	**0.001**	1.698(1.298-2.222)	**<0.001**	1.566(1.185-2.072)	**0.002**
PLR(>166.1)	1.937(1.326-2.828)	**0.001**	1.600(1.052-2.234)	**0.028**	1.840(1.413-2.395)	**<0.001**	-	-
dNLR(>3.7)	1.932(1.323-2.822)	**0.001**	-	-	2.051(1.585-2.655)	**<0.001**	1.726(1.294-2.303)	**<0.001**
NLR (>2.9)	2.023(1.382-2.959)	**<0.001**	-	-	1.995(1.532-2.598)	**<0.001**	-	-
LMR(>1.5)	0.437(0.221-0.867)	**0.018**	-	-	0.374(0.253-0.553)	**<0.001**	-	-
Fib (>3.9 g/L)	3.165(2.023-4.954)	**<0.001**	1.934(1.153-3.243)	**0.012**	1.917(1.403-2.619)	**<0.001**	1.428(1.017-2.007)	**0.040**
Alb (>35.7 g/L)	0.461(0.289-0.735)	**0.001**	0.603(0.370-0.983)	**0.042**	0.511(0.384-0.681)	**<0.001**	0.716(0.522-0.982)	**0.038**
pAlb (>139.8mg/L)	0.444(0.246-0.801)	**0.007**	-	**-**	0.414(0.266-0.644)	**<0.001**	0.517(0.322-0.831)	**0.006**
AFR (>9.9)	0.344(0.219-0.541)	**<0.001**	0.568(0.340-0.951)	**0.032**	0.453(0.332-0.618)	**<0.001**	-	**-**
FPR (>22.8)	3.150(1.708-5.810)	**<0.001**	2.254(1.167-4.354)	**0.015**	2.984(1.874-4.753)	**<0.001**	1.769(1.066-2.935)	**0.027**

Abbreviation: HR: hazard ratio; CI: confidence interval; Fib: fibrinogen; Alb: albumin; pAlb: pre-Albumin; AFR: albumin to fibrinogen ratio; FPR: fibrinogen to pre-Albumin ratio; PLR: Platelet to lymphocyte ratio, dNLR: derived neutrophil to lymphocyte ratio; NLR: Neutrophil granulocyte to lymphocyte ratio; LMR: lymphocyte to monocyte ratio; CEA: carcinoembryonic antigen; CA199: carbohydrate antigen 19-9; adjusted HR (95%) was adjusted by sex, age, alcohol, tobacco, hypertension, diabetes, palliative resection, radio-chemotherapy, CEA, CA199, PLR, dNLR, NLR, LMR.

Supplementary Table 4. Univariate and multivariate analyses of prognostic factors for 3 years' PFS and OS by Cox regression model in right-sided mCRC patient in discovery cohort.

Variables	Progression-free survival				Overall survival			
	Univariate analysis		Multivariate analysis		Univariate analysis		Multivariate analysis	
	Crude HR(95% CI)	*p*-value	Adjusted HR(95% CI)	*p*-value	Crude HR(95% CI)	*p*-value	Adjusted HR(95% CI)	*p*-value
Sex (male)	1.895(1.031-3.483)	**0.039**	2.380(1.212-4.674)	**0.012**	1.445(0.990-2.109)	0.057	-	-
Age (>60 years)	0.762 (0.418-1.390)	0.376	-	-	1.069(0.734-1.558)	0.728	-	-
Alcohol (yes)	0.743(0.229-2.410)	0.621	-	-	1.128(0.654-1.948)	0.664	-	-
Tobacco (yes)	0.937(0.334-2.631)	0.902	-	-	1.196(0.712-2.008)	0.499	-	-
Hypertension (yes)	0.573(0.241-1.361)	0.207	-	-	0.662(0.370-1.183)	0.164	-	-
Diabetes (yes)	1.095(0.487-2.464)	0.826	-	-	0.665(0.323-1.369)	0.269	-	-
Palliative resection (Yes)	0.699(0.378-1.292)	0.254	-	-	0.493(0.336-0.723)	**<0.001**	0.351(0.227-0.543)	**<0.001**
Radio-chemotherapy (Yes)	0.832(0.419-1.653)	0.600	-	-	0.522(0.356-0.766)	**0.001**	0.424(0.277-0.648)	**<0.001**
CEA (>5ng/mL)	1.701(0.779-3.714)	0.182			1.747(1.052-2.901)	0.031	-	-
CA199 (>37U/mL)	2.750(1.362-5.554)	**0.005**	3.110(1.517-6.376)	**0.002**	1.726(1.138-2.619)	**0.010**	1.829(1.194-2.801)	**0.006**
PLR(>166.1)	1.812 (0.523-2.286)	0.812	-	-	0.866(0.546-1.372)	0.539	-	-
dNLR(>3.7)	1.008(0.546-1.859)	0.980	-	-	1.265(0.868-1.844)	0.221	-	-
NLR (>2.9)	1.305(0.712-2.393)	0.389	-	-	1.287(0.867-1.909)	0.210	-	-
LMR(>1.5)	0.834(0.298-2.339)	0.731	-	-	0.737(0.426-1.273)	0.273	-	-
Fib (>3.9 g/L)	1.146(0.603-2.176)	0.677	-	-	1.024(0.668-1.570)	0.912	-	-
Alb (>35.7 g/L)	1.713 (0.818-3.589)	0.153	-	-	1.075(0.724-1.595)	0.720	-	-
pAlb (>139.8mg/L)	0.613(0.252-1.492)	0.281	-	-	0.511(0.257-1.014)	0.055	-	-
AFR (>9.9)	1.256 (0.664-2.377)	0.458	-	-	1.049(0.684-1.608)	0.828	-	-
FPR (>22.8)	1.265(0.516-3.102)	0.607	-	-	1.904(0.906-4.002)	0.089	-	-

Abbreviation: HR: hazard ratio; CI: confidence interval; Fib: fibrinogen; Alb: albumin; pAlb: pre-Albumin; AFR: albumin to fibrinogen ratio; FPR: fibrinogen to pre-Albumin ratio; PLR: Platelet to lymphocyte ratio, dNLR: derived neutrophil to lymphocyte ratio; NLR: Neutrophil granulocyte to lymphocyte ratio; LMR: lymphocyte to monocyte ratio; CEA: carcinoembryonic antigen; CA199: carbohydrate antigen 19-9; adjusted HR (95%) was adjusted by sex, age, alcohol, tobacco, hypertension, diabetes, palliative resection, radio-chemotherapy, CEA, CA199, PLR, dNLR, NLR, LMR.

Supplementary Table 5. Univariate and multivariate analyses of prognostic factors for 3 years' PFS and OS by Cox regression model in validation cohort.

Variables	Progression-free survival				Overall survival			
	Univariate analysis		Multivariate analysis		Univariate analysis		Multivariate analysis	
	**Crude HR(95% CI)**	***p*-value**	**Adjusted HR(95% CI)**	***p*-value**	**Crude HR(95% CI)**	***p*-value**	**Adjusted HR(95% CI)**	***p*-value**
Sex (male)	1.051(0.631-1.752)	0.847	-	-	1.029(0.568-1.865)	0.925	-	-
Age (>60 years)	1.024(0.594-1.765)	0.932	-	-	0.957(0.376-2.435)	0.926	-	-
Alcohol (yes)	0.559(0.223-1.401)	0.214	-	-	0.950(0.374-2.414)	0.914	-	-
Tobacco (yes)	0.872 (0.430-1.772)	0.706	-	-	1.379(0.681-2.793)	0.372	-	-
Hypertension (yes)	1.467(0.777-2.767)	0.237	-	-	1.840(0.926-3.657)	0.082		
Diabetes (yes)	1.593 (0.491-5.173)	0.438	-	-	2.651(0.933-7.533)	0.067		
Primary side(Right)	0.608(0.355-1.041)	0.070	-	-	0.718(0.388-1.330)	0.292		
Palliative resection (Yes)	0.688(0.415-1.139)	0.146	-	-	0.368(0.197-0.688)	**0.002**	0.328(0.160-0.670)	**0.002**
CEA (>5ng/mL)	1.616(0.859-3.037)	0.136	-	-	2.433(1.127-5.252)	**0.024**	3.242(1.385-7.586)	**0.007**
CA199 (>37U/mL)	1.244(0.689-2.244)	0.469	-	-	1.604(0.798-3.224)	0.185	-	-
PLR(>166.1)	1.724(1.021-2.911)	**0.042**	-	-	1.540(0.840-2.826)	0.163	-	-
dNLR(>3.7)	1.518(0.914-2.521)	0.107	-	-	1.750(0.962-3.185)	0.067	-	-
NLR (>2.9)	1.466(0.884-2.432)	0.138	-	-	1.536(0.842-2.801)	0.162	-	-
LMR(>1.5)	0.965(0.437-2.129)	0.930	-	-	1.274(0.455-3.568)	0.644	-	-
Fib (>3.9 g/L)	1.474(0.878-2.474)	0.142	-	-	2.233(1.234-4.414)	**0.009**	2.498(1.191-5.239)	**0.015**
Alb (>35.7 g/L)	0.977(0.506-1.886)	0.945	-	-	0.593(0.302-1.164)	0.129	-	-
pAlb (>139.8mg/L)	0.627(0.337-1.167)	0.141	-	-	0.397(0.176-0.897)	**0.026**	-	-
AFR (>9.9)	0.698(0.412-1.184)	0.182	-	-	0.569(0.302-1.072)	0.081	-	-
FPR (>22.8)	1.914(1.022-3.583)	**0.042**	-	-	4.473(1.648-12.141)	**0.003**	4.206(1.159-15.266)	**0.029**

Abbreviation: HR: hazard ratio; CI: confidence interval; Fib: fibrinogen; Alb: albumin; pAlb: pre-Albumin; AFR: albumin/fibrinogen ratio; FPR: fibrinogen/pre-Albumin ratio; PLR: Platelet/lymphocyte ratio, dNLR: derived neutrophil to lymphocyte ratio; NLR: Neutrophil granulocyte/lymphocyte ratio; LMR: lymphocyte/monocyte ratio; CEA: carcinoembryonic antigen; CA199: carbohydrate antigen 19-9; adjusted HR (95%) was adjusted by sex, age, alcohol, tobacco, hypertension, diabetes, primary side, palliative resection, CEA, CA199, PLR, dNLR, NLR, LMR.

Supplementary Table 6. Univariate and multivariate analyses of prognostic factors for 3 years' PFS and OS by Cox regression model in left-sided mCRC patient in validation cohort.

Variables	Progression-free survival				Overall survival			
	Univariate analysis		Multivariate analysis		Univariate analysis		Multivariate analysis	
	**Crude HR(95% CI)**	***p*-value**	**Adjusted HR(95% CI)**	***p*-value**	**Crude HR(95% CI)**	***p*-value**	**Adjusted HR(95% CI)**	***p*-value**
Sex (male)	1.286(0.647-2.556)	0.347	-	-	0.964(0.457-2.035)	0.924	-	-
Age (>60 years)	1.359(0.520-3.551)	0.532	-	-	1.513(0.641-3.572)	0.345	-	-
Alcohol (yes)	0.744(0.225-1.461)	0.628	-	-	1.430(0.428-4.778)	0.561	-	-
Tobacco (yes)	1.386 (0.596-3.226)	0.448	-	-	1.871(0.821-4.265)	0.136	-	-
Hypertension (yes)	0.548(0.186-1.612)	0.275	-	-	1.045(0.396-2.758)	0.929	-	-
Diabetes (yes)	1.607 (0.376-6.870)	0.522	-	-	3.382(0.969-11.805)	0.056	-	-
Palliative resection (Yes)	0.387(0.186-0.803)	**0.011**	0.186(0.064-0.542)	**0.002**	0.389(0.179-0.844)	**0.017**	0.421(0.181-0.977)	**0.044**
CEA (>5ng/mL)	1.344(0.506-3.568)	0.553	-	-	2.049(0.746-5.626)	0.164	-	-
CA199 (>37U/mL)	1.139(0.519-2.150)	0.745	-	-	1.196(0.528-2.711)	0.668	-	-
PLR(>166.1)	0.868(0.440-1.713)	0.683	-	-	1.111(0.527-2.341)	0.781	-	-
dNLR(>3.7)	1.089(0.529-2.241)	0.818	-	-	1.237(0.583-2.624)	0.580	-	-
NLR (>2.9)	1.221(0.610-2.445)	0.574	-	-	1.060(0.503-2.233)	0.878	-	-
LMR(>1.5)	2.936(0.689-12.506)	0.145	-	-	1.831(0.433-7.736)	0.411	-	-
Fib (>3.9 g/L)	1.348(0.675-2.693)	0.348	-	-	2.616(1.092-6.265)	**0.031**	2.960(1.071-8.180)	**0.036**
Alb (>35.7 g/L)	1.431(0.544-3.768)	0.468	-	-	0.387(0.164-0.915)	**0.031**	0.115(0.028-0.467)	**0.003**
pAlb (>139.8mg/L)	0.534(0.222-1.283)	0.160	-	-	0.382(0.127-1.148)	0.087	-	-
AFR (>9.9)	0.735(0.361-1.497)	0.397	-	-	0.520(0.227-1.190)	0.122	-	-
FPR (>22.8)	1.420(0.616-3.276)	0.410	-	-	4.112(1.126-15.015)	**0.032**	-	-

Abbreviation: HR: hazard ratio; CI: confidence interval; Fib: fibrinogen; Alb: albumin; pAlb: pre-Albumin; AFR: albumin/fibrinogen ratio; FPR: fibrinogen/pre-Albumin ratio; PLR: Platelet/lymphocyte ratio, dNLR: derived neutrophil to lymphocyte ratio; NLR: Neutrophil granulocyte/lymphocyte ratio; LMR: lymphocyte/monocyte ratio; CEA: carcinoembryonic antigen; CA199: carbohydrate antigen 19-9; adjusted HR (95%) was adjusted by sex, age, alcohol, tobacco, hypertension, diabetes, palliative resection, CEA, CA199, PLR, dNLR, NLR, LMR.

Supplementary Table 7. Univariate and multivariate analyses of prognostic factors for 3 years' PFS and OS by Cox regression model in right-sided mCRC patient in validation cohort.

Variables	Progression-free survival				Overall survival			
	Univariate analysis		Multivariate analysis		Univariate analysis		Multivariate analysis	
	Crude HR(95% CI)	*p*-value	Adjusted HR(95% CI)	*p*-value	Crude HR(95% CI)	*p*-value	Adjusted HR(95% CI)	*p*-value
Sex (male)	0.783(0.334-1.836)	0.574	-	-	1.059(0.384-1.914)	0.912	-	-
Age (>60 years)	1.154(0.516-2.581)	0.728	-	-	0.713(0.259-1.967)	0.514	-	-
Alcohol (yes)	0.471(0.110-2.018)	0.628	-	-	0.757(0.172-3.140)	0.714	-	-
Tobacco (yes)	0.471 (0.110-.018)	0.310	-	-	0.757(0.172-3.140)	0.714	-	-
Hypertension (yes)	3.233(1.156-9.042)	**0.025**	-	-	3.506(1.265-9.715)	**0.016**	-	-
Diabetes (yes)	1.664 (0.216-12.822)	0.625	-	-	1.523(0.198-11.701)	0.686	-	-
Palliative resection (Yes)	0.924(0.414-2.060)	0.847	-	-	0.241(0.077-0.750)	**0.014**	-	-
CEA (>5ng/mL)	1.639(0.636-4.227)	0.306	-	-	2.282 (0.697-7.466)	0.173	-	-
CA199 (>37U/mL)	1.549(0.582-4.123)	0.381	-	-	3.287(0.726-14.882)	0.123	-	-
PLR(>166.1)	3.892(1.508-10.047)	**0.005**	3.158(1.086-9.182)	**0.035**	2.283(0.773-6.747)	0.135	-	-
dNLR(>3.7)	2.430(1.012-5.836)	**0.047**	-	-	3.584(1.129-11.376)	**0.030**	-	-
NLR (>2.9)	2.054(0.856-4.930)	0.107	-	-	3.025(0.948-9.649)	0.061	-	-
LMR(>1.5)	0.435(0.136-1.396)	0.162	-	-	0.890(0.201-3.952)	0.879	-	-
Fib (>3.9 g/L)	1.859(0.781-4.425)	0.161	-	-	2.106 (0.761-5.828)	0.152	-	-
Alb (>35.7 g/L)	0.696(0.251-1.926)	0.485	-	-	0.930(0.299-2.890)	0.900	-	-
pAlb (>139.8mg/L)	0.782(0.286-2.140)	0.632	-	-	0.354(0.094-1.333)	0.125	-	-
AFR (>9.9)	0.580(0.243-1.385)	0.220	-	-	0.642(0.232-1.775)	0.393	-	-
FPR (>22.8)	2.845(0.940-8.615)	0.064	-	-	4.538(0.938-21.958)	0.060	-	-

Abbreviation: HR: hazard ratio; CI: confidence interval; Fib: fibrinogen; Alb: albumin; pAlb: pre-Albumin; AFR: albumin/fibrinogen ratio; FPR: fibrinogen/pre-Albumin ratio; PLR: Platelet/lymphocyte ratio, dNLR: derived neutrophil to lymphocyte ratio; NLR: Neutrophil granulocyte/lymphocyte ratio; LMR: lymphocyte/monocyte ratio; CEA: carcinoembryonic antigen; CA199: carbohydrate antigen 19-9; adjusted HR (95%) was adjusted by sex, age, alcohol, tobacco, hypertension, diabetes, palliative resection, CEA, CA199, PLR, dNLR, NLR, LMR.
